# Protective Activity and Underlying Mechanism of Ginseng Seeds against UVB-Induced Damage in Human Fibroblasts

**DOI:** 10.3390/antiox10030403

**Published:** 2021-03-08

**Authors:** Huijin Heo, Hana Lee, Jinwoo Yang, Jeehye Sung, Younghwa Kim, Heon Sang Jeong, Junsoo Lee

**Affiliations:** 1 Department of Food Science and Biotechnology, Chungbuk National University, Cheongju, Chungbuk 28644, Korea; pltreasure11@gmail.com (H.H.); dlgksk0514@naver.com (H.L.); hsjeong@chungbuk.ac.kr (H.S.J.); 2 Wheat Research Team, National Institute of Crop Science, Rural Development Adminstration, Wanju, Jeonbuk 55365, Korea; jinwoo1127@korea.kr; 3 Department of Food Science and Biotechnology, Andong National University, Andong, Gyeongbuk 36729, Korea; jeehye@anu.ac.kr; 4 School of Food Biotechnology and Nutrition, Kyungsung University, Busan 48434, Korea; younghwakim@ks.ac.kr

**Keywords:** ginseng seeds, human skin fibroblast, MAPK, photoaging, UVB

## Abstract

Ginseng seeds are rich in phytosterols, ginsenosides, and fatty acids, and can therefore be used in skincare to delay the aging process. Ginseng seed embryo (GSE) and ginseng seed coat (GSC) were separated from ginseng seeds (Panax ginseng Meyer). This study evaluated the protective activity and underlying mechanism of GSE and GSC on UVB irradiation-induced skin photoaging using Hs68 cells. Their bioactive compounds, including phytosterols, ginsenosides, tocopherols, tocotrienols, and fatty acids were determined by HPLC and GC. The levels of reactive oxygen species, matrix metalloproteinases (MMPs), and collagen levels were measured in human dermal fibroblast cell line, Hs68 cells. The antioxidant capacity and contents of total polyphenols and flavonoids were higher in GSC than those in GSE. Linoleic acid was the major fatty acid in both GSE and GSC. GSE and GSC treatment alleviated UVB-induced increase of reactive oxygen species (ROS), matrix metalloproteinase (MMP)-1, and MMP-3, resulting in reduced collagen degradation. Increased UVB-mediated phosphorylation of mitogen activated protein kinase (MAPK) and activator protein-1 (AP-1) was inhibited by GSE and GSC treatment. Moreover, GSE and GSC effectively upregulated transforming growth factor-β (TGF-β) 1 levels. It was found that ginseng seeds regulate the expression of TGF-β/Smad and MAPK/AP-1 pathways. Ginseng seeds contain various bioactive compounds and have protective activity against UVB-induced skin photoaging. Therefore, ginseng seeds have the potential for use in cosmeceutical preparations.

## 1. Introduction

UVB radiation causes deleterious effects on human skin, including photoaging, sunburn, and cancer [1]. Fibroblasts are the main cellular component of skin for the metabolism of extracellular matrix (ECM) proteins, including procollagen, elastin, and fibronectin [2,3]. UVB irradiation increases reactive oxygen species (ROS) production and stimulates matrix metalloproteinases (MMPs) expression in the fibroblasts, resulting in collagen degradation [4]. MMPs are regulated by the mitogen activated protein kinase (MAPK) family members and activator protein-1 (AP-1) transcriptional factors [5,6]. Transforming growth factor-β (TGF-β) binds to the specific receptor and induces the phosphorylation of Smad 2/3 protein [1,7]. Phosphorylated Smad 2/3 moves into the nucleus and promotes procollagen transcription [8]. Smad 7 was found to negatively regulate the TGF-β/Smad expression by inhibiting the activation of Smad 2/3 [9]. UVB irradiation inhibits procollagen synthesis by modulating the TGF-β/Smad pathways [10]. Thus, agents that modulate collagen synthesis and degradation signaling pathway may be beneficial for preventing and treating skin photoaging.

Ginseng root (Panax ginseng C. A. Meyer) is one of the most widely used traditional herbs for health [11]. Previous studies have consistently shown that ginseng root provides a rich source of phytochemicals including saponins (ginsenosides), polysaccharides, polyacetylenes, and phenolic compounds that exhibit diverse health benefits [12,13,14]. In recent years, it has been noted that other parts or processing by-products of the ginseng plant such as leaves, berries, and flower buds also have potential therapeutic activities [15,16,17]. Ginseng seeds are used to cultivate ginseng and recent studies have shown that ginseng seeds contain biologically active compounds similar to those in ginseng roots [18]. Ginseng seeds contain functional compounds, such as fatty acids [19], phytosterols [20], and saponins [18]. These compounds in the ginseng seeds have been reported to prevent skin aging [21,22,23]. However, there is little evidence regarding the anti-photoaging activity of ginseng seeds against UVB-induced damage. Therefore, this study evaluated the protective activities and underlying mechanisms of ginseng seeds on UVB-induced photoaging in fibroblasts. For the underlying mechanisms, we evaluated the effects of ginseng seeds on ROS formation, MMP production, total collagen production, and regulation of MAPK, AP-1, TGF-β, and Smad expressions.

## 2. Materials and Methods

### 2.1. Reagents

Trolox, sodium carbonate (Na_2_CO_3_), Folin-Ciocalteu reagent, gallic acid, sodium nitrite (NaNO_2_), dimethyl sulfoxide (DMSO), aluminum trichloride (AlCl3), sodium hydroxide (NaOH), and catechin were obtained from Sigma Chemical Co. (St. Louis, MO, USA). Phospho-c-fos (p-c-fos), c-fos, phospho-c-jun (p-c-jun), c-jun, TGF-β1, and β-actin were purchased from Santa Cruz Biotechnology (CA, USA). Antibodies against phospho-ERK (p-ERK), ERK, phospho-JNK (p-JNK), JNK, phospho-p38 (p-p38), and p38 were obtained from Cell Signaling Technology (Beverly, MA, USA). Phospho-Smad 2/3 (p-Smad 2/3) and Smad 2/3 antibodies obtained from Abcam (Cambridge, UK).

### 2.2. Preparation of Ginseng Seeds Extract

Ginseng seeds (Panax ginseng Meyer) were obtained from a local market (Geumsan, Korea) in 2019. The seeds were separated into ginseng seed embryo (GSE) and ginseng seed coat (GSC). GSE and GSC (20 g each) were extracted with 400 mL of methanol for 24 h using a shaker at 23 °C. The extracts were filtered and then concentrated under vacuum at 37 °C. The concentrates were dissolved in DMSO, filtered through a 0.22 μm sterile filter, and stored at −20 °C before use.

### 2.3. Antioxidant Activities

Free radical scavenging capacities of GSE and GSC were measured using DPPH and ABTS radicals, according to the methods of Cheung et al. [24] and Re et al. [25], respectively. The total polyphenol content (TPC) and total flavonoid content (TFC) in GSE and GSC were assayed using the method of Choi et al. [26] and Sung et al. [27], respectively.

### 2.4. Phytosterols, Tocopherols, and Tocotrienols

GSE and GSC extracts were saponified by refluxing with 20 mL of 6% *(w*/*v)* ethanolic pyrogallol and 8 mL of 60% potassium hydroxide aqueous solution for 50 min at 70 °C. The saponified samples were cooled in an ice bath, and 30 mL of 2% sodium chloride was added. The suspension was then extracted three times with a 20 mL portion of n-hexane:ethyl acetate (85:15, *v*/*v*) with 0.1% butylated hydroxytoluene (BHT). The organic layer was filtered through anhydrous sodium sulfate and then evaporated at 37 °C. Finally, the extracts were dissolved in chloroform or hexane to determine the phytosterols and vitamin E contents, respectively.

The contents of phytosterols in GSE and GSC extracts were assayed according to the method of Choi et al. [28]. GC (Varian 3800; Varian Inc., Walnut Creek, CA, USA) was equipped with an SAC-5 fused-silica capillary column (30 m × 60.32 mm i.d.; Supelco, Bellefonte, PA, USA) and a flame ionization detector. The column was held at 280 °C for 1 min and programmed to rise to 300 °C at a rate of 2 °C/min. It was then held at 300 °C for 20 min. The carrier gas was helium, and the total gas flow rate was 20 mL/min. The injector and detector temperatures were 310 °C and 320 °C, respectively. Comparison of the retention times with those of the standards permitted the identification of the sterols, squalene, and octacosanol. The chromatograms of phytosterols were shown in (Supplementary Materials Figure S1).

The contents of tocopherols and tocotrienols in GSE and GSC extracts were assayed according to the method of Sim et al. [29]. Tocopherols and tocotrienols were analyzed by using an HPLC system equipped with a PU-2089 pump, an AS-2059 auto injector, and an FP-2020 fluorescence detector (JASCO Crop.). The separation was carried on a LiChrospher^®^ column (250 × 4 m^2^, 5 μm i.d.; Merck, Berlin, Germany) with isocratic elution in a mobile phase of hexane/isopropanol (98.9:1.1, *v*/*v*). An excitation wavelength of 290 nm and emission wavelength of 330 nm were used to detect the peaks. The chromatograms of tocopherols and tocotrienols were shown in (Supplementary Materials Figure S2).

### 2.5. Ginsenosides

The amounts of ginsenosides in GSE and GSC extracts were assayed according to the method of Jang et al. [30]. GSE and GSC extracts were subjected to open-column chromatography on a Diaion HP-20 (Mitsubishi Chemical Co., Ltd., Tokyo, Japan) and washed repeatedly with distilled water. The ginsenoside bound to Diaion HP-20 resin was recovered by elution with methanol. The methanol fraction was evaporated at 37 °C and dissolved in methanol again to prepare them for HPLC analysis. The analysis of the ginsenosides was performed using an HPLC system (Agilent Technology Inc., Wilmington, NC, USA) equipped with a quaternary pump, automatic injector, and wavelength UV detector (1200 Series). The separation was carried out on a Mightytsil RP-18 GP column (250 × 4.6 mm^2^, 2.6 μm; Kanto Chemical Co., Inc., Tokyo, Japan). The mobile phase of the analytical system consisted of acetonitrile (A) and water (B) operating under the following gradient with mobile 0–42 min (18–24% A), 42–46 min (24–29% A), 46–75 min (29–40% A), 75–100 min (40–65% A), 100–135 min (65–85% A), and 135–150 min (85% A). The flow rate was set at 0.6 mL/min and the injection volume was 20 μL. Detection was performed by monitoring the absorbance at 203 nm. The chromatograms of ginsenosides were shown in (Supplementary Materials Figure S3).

### 2.6. Fatty Acids

The amounts of fatty acids in GSE and GSC extracts were assayed according to the method of Duan et al. [31]. Fatty acids in GCE and GSC extracts were separated as methyl esters of fatty acids by BF3-catalyzed transesterification and quantified with a Hewlett-Packard 6890 gas chromatograph (Santa Clara, CA, USA) equipped with an SPTM-2560 capillary column (biscyanopropyl polysiloxane, 100 m × 0.25 mm, 0.25 μm film thickness, Supelco, Bellefonte, PA, USA), and a flame ionized detector (Hewlett-Packard, Avondale, PA, USA), as previously described. The chromatograms of fatty acids were shown in (Supplementary Materials Figure S4).

### 2.7. Cell Viability and UVB Irradiation

Hs68 (ATCC, CRL-1635, Manassas, VA, USA) cells were grown in DMEM containing 100 unit/mL penicillin, 10% heat-inactivated FBS, and 100 μg/mL streptomycin in 5% CO_2_ humidified air at 37 °C. The cells were seeded at a density of 1 × 104 cells/well in 96-well plates for 24 h and then pretreated with GSE, GSC, or ascorbic acid (ASA) for 24 h. The culture medium was changed with phosphate-buffered saline (PBS), and the cells were treated with 30 mJ/cm² UVB followed by additional 24 h incubation in FBS-free medium (DMEM containing 100 unit/mL penicillin and 100 μg/mL streptomycin) containing the same concentration of GSE, GSC, or ASA. An MTT assay was used to assess cell viability [32].

### 2.8. Measurement of ROS

Hs68 cells were plated at a density of 1 × 10^4^ cells/well in black 96-well plates for 24 h. The cells were pretreated with GSE, GSC, or ASA for 24 h, washed twice with PBS, and then exposed to 30 mJ/cm² UVB irradiation. After irradiation, the cells were treated for an additional 30 min in FBS-free medium containing GSE, GSC, or ASA and incubated with 25 μM DCFH-DA for 30 min. The fluorescence intensity at 485 and 530 nm for excitation and emission wavelength was determined.

### 2.9. Measurement of MMPs and Total Collagen

MMP production and total collagen synthesis were measured using an enzyme-linked immunosorbent assay (ELISA) kit (QIA55; Merck & Co., Inc., Whitehouse Station, NJ, USA) and a Sircol^TM^ assay kit (Biocolor Ltd., Newtownabbey, UK), respectively.

### 2.10. Western Blot Analysis

The protein concentration in whole cell extracts was measured using the BCA protein assay reagent. The proteins were separated on SDS-polyacrylamide gels, transferred to nitrocellulose membranes, and the membrane blocked with 5% nonfat milk in Tris-buffered saline and Tween 20 (TBST) for 1 h and incubated with primary antibodies at 4 °C. After washing with TBST buffer, the membranes were incubated with the corresponding secondary antibodies at room temperature for 1 h. Protein bands were activated by chemiluminescence using the SuperSignal West Pico chemiluminescent substrate and visualized on X-ray film. The band intensities were quantified using Image J software (NIH, Bethesda, MD, USA).

### 2.11. Statistical Analysis

Analytical data are shown as the mean ± standard error (SE). All cell-based experiments were performed in triplicates. ANOVA with the Tukey’s post-hoc test was used (SAS Institute, Cary, NC, USA).

## 3. Results and Discussion

### 3.1. Antioxidant Activities and Phytochemical Content

Oxidative stress reduces the antioxidant capacity of cells and increases ROS generation, thereby resulting in skin aging [33]. Antioxidant compounds, such as vitamin E, vitamin C, polyphenols, and flavonoids, are main antioxidants in the skin [34]. The antioxidant compounds, antioxidant activities, and ginsenoside content of GSC were superior to those of GSE, whereas the phytosterols, tocopherols, tocotrienol, and fatty acid content of GSE were higher than those of GSC (Table 1). The campesterol content was similar between GSE (6.35 mg/g residue) and GSC (6.11 mg/g residue). The stigmasterol and β-sitosterol contents of GSE were 5.3 and 5.1 times higher than that of GSC, respectively. Ginsenoside Rh4 and Re is the major ginsenoside in GSE and GSC. It was reported that ginsenoside Rh4 content in GSC was higher than in Panax ginseng root [35]. α-tocopherol was detected in only GSE, and the α-tocotrienol content of GSE (202.97 μg/g residue) was the highest among the total vitamin E contents. β- and δ-tocopherols and β-, γ-, and δ-tocotrienols were not detected in ginseng seeds. The major fatty acids of GSE and GSC were linoleic acid (58% and 40%, respectively), followed by oleic acid (20% and 34%, respectively) and palmitic acid (16% and 11%, respectively). These results indicate that GSC showed higher antioxidant activities compared to GSE due to higher contents of TPC and TFC. In previous reports, antioxidant activities of nuts and millet with coat were higher than those without coat [36,37]. On the other hand, GSE contains higher amounts of lipid-soluble compounds including phytosterols, tocopherols, tocotrienols, and fatty acids than GSC because they are oily. Ginsenoside could be used in cosmetic products to protect the skin from UVB rays and increase the moisture levels in the skin [22]. Tocotrienols protect the skin more strongly against UVB-induced damage than α-tocopherol [38]. Fatty acids reduce UV-induced inflammation in the skin, in addition to potentially offering protection against photoaging, photocarcinogenesis, and photosensitivity disorders [21]. Therefore, these results imply that GSC and GSE containing these active compounds may prevent skin aging.

### 3.2. Protective Effect against UVB-Induced Damage

**Figure 1:**
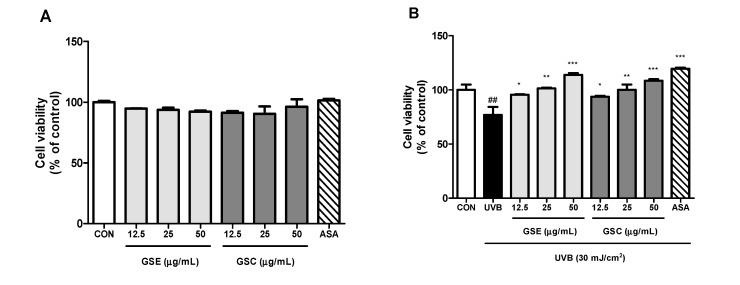
Effects of ginseng seed embryo (GSE) and coat (GSC) on Hs68 cells. (**A**) Cytotoxic effect of GSE and GSC on Hs68 cells was evaluated using MTT assay. (**B**) Protective effect of GSE and GSC in UVB-irradiated Hs68 cells was evaluated using MTT assay. Values are expressed as the mean ± standard error (*n* = 3). ## *p* < 0.01, significant difference compared to control; *, **, and *** *p* < 0.05, 0.01, and 0.001, respectively, significant difference compared to UVB-irradiated group. ASA (ascorbic acid, 100 μM), positive control.

To evaluate the cytotoxic effects of GSE and GSC on Hs68 cells, an MTT assay was performed. Treatment of Hs68 cells with GSE and GSC up to concentration of 50 μg/mL for 48 h had no cytotoxic effect (Figure 1A). UVB (30 mJ/cm^2^) irradiation significantly suppressed the cell viability (76.9%) compared to that of control cells (100%) (Figure 1B). GSE and GSC protected dermal fibroblasts cells from UVB-induced phototoxicity in a dose-dependent manner.

### 3.3. Measurement of ROS, MMPs, and Collagen Production

**Figure 2:**
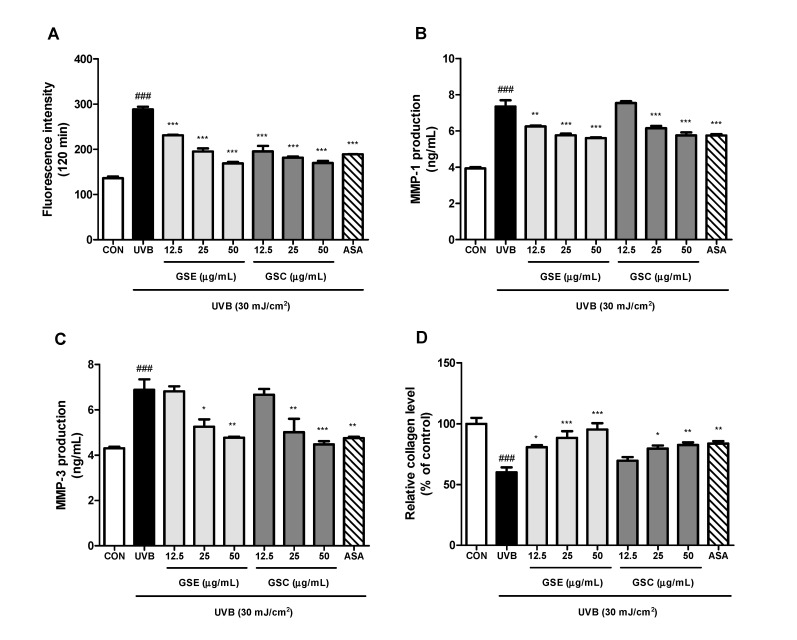
Effects of ginseng seed embryo (GSE) and coat (GSC) on reactive oxygen species (ROS), matrix metalloproteinases (MMPs), and collagen production in UVB-irradiated Hs68 cells. Control or UVB-irradiated cells were treated with/without various concentration of GSE and GSC and then analyzed for (**A**) ROS using the DCFH-DA fluorogenic dye, (**B**) MMP-1 using ELISA kit, (**C**) MMP-3 using ELISA kit, and (**D**) collagen production using Sicrol^TM^ assay kit. Values are expressed as the mean ± standard error (*n* = 3). ### *p* < 0.001, significant difference compared to control; *, **, and *** *p* < 0.05, 0.01, and 0.001, respectively, significant difference compared to UVB-irradiated group. ASA (ascorbic acid, 100 μM), positive control.

UVB induces the production of ROS, which damages cellular components. ROS triggers the expression of MMPs that enhance the degradation of ECM proteins, such as collagen [39]. ROS production was significantly higher in UVB-irradiated cells compared to control cells; however, the increased ROS production was attenuated in a concentration-dependent manner in UVB-irradiated cells treated with GSE and GSC extract (12.5–50 μg/mL) (Figure 2A). MMP-1 mainly degrades fibrillar collagens of the skin, whereas MMP-3 degrades a variety of ECM substrates and activates other secreted MMPs [39,40]. Production of MMP-1 (Figure 2B) and MMP-3 (Figure 2C) were significantly reduced in the GSE- and GSC-treated cells. In addition, GSE and GSC treatment restored collagen production in UVB-irradiated Hs68 cells compared with untreated UVB-irradiated cells in a concentration-dependent manner (Figure 2D). The extent of MMP inhibition and collagen regeneration at 50 μg/mL GSE and GSC treatment were similar to those of ascorbic acid as a positive control.

### 3.4. Phosphorylation of MAPK and AP-1

**Figure 3:**
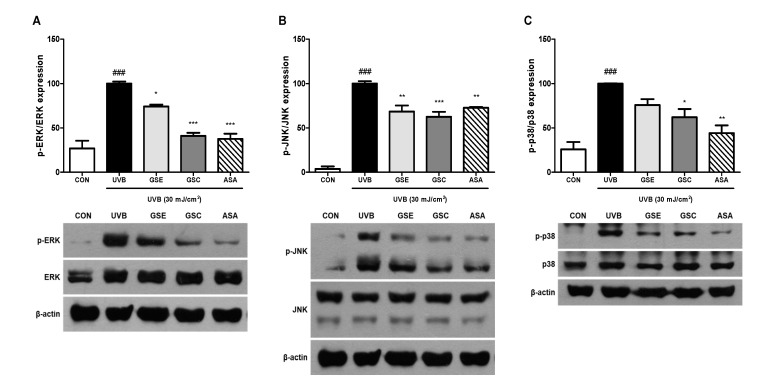
Effects of ginseng seed embryo (GSE) and coat (GSC) on the phosphorylation of mitogen activated protein kinase (MAPK) proteins in UVB-irradiated Hs68 cells. Control or UVB-irradiated cells were treated with/without GSE or GSC and subjected to western blotting for (**A**) p-ERK, (**B**) p-JNK, and (**C**) p-p38 levels. The relative levels of the phosphorylated proteins were normalized to those of the respective total MAPK proteins. Values are expressed as the mean ± standard error (*n* = 3). ### *p* < 0.001, significant difference compared to control; *, **, and *** *p* < 0.05, 0.01, and 0.001, respectively, significant difference compared to UVB-irradiated group. ASA (ascorbic acid, 100 μM), positive control.

**Figure 4:**
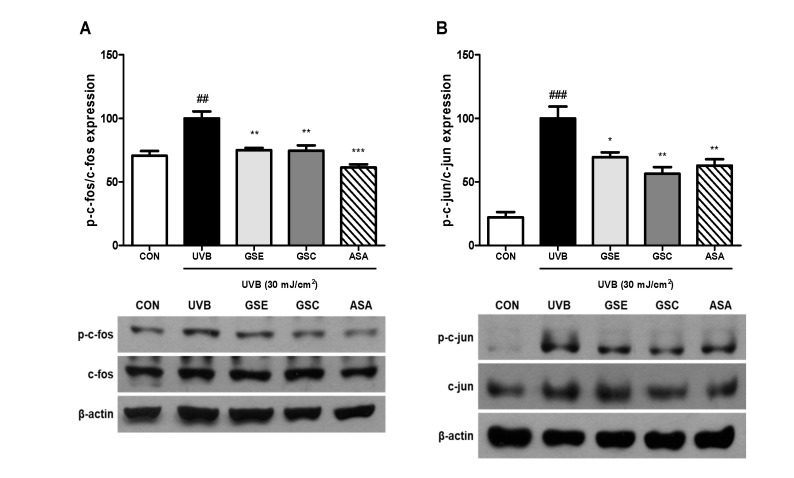
Effects of ginseng seed embryo (GSE) and coat (GSC) on the phosphorylation of activator protein-1 (AP-1) proteins in UVB-irradiated Hs68 cells. Control or UVB-irradiated cells were treated with/without GSE or GSC and subjected to western blotting for (**A**) p-c-fos and (**B**) p-c-jun levels. The relative levels of phosphorylated protein were normalized to those of total AP-1 proteins. Values are expressed as the mean ± standard error (*n* = 3). ## and ### *p* < 0.01 and 0.001, significant difference compared to control; *, **, and *** *p* < 0.05, 0.01, and 0.001, respectively, significant difference compared to UVB-irradiated group. ASA (ascorbic acid, 100 μM), positive control.

MAPK phosphorylation is involved in photoaging and MMP production in fibroblasts [1]. Three main MAPK pathway, including ERK, JNK, and p38 have been reported to increase MMP production by activating the AP-1 transcription factors [5,6]. GSE and GSC treatment reduced UVB-mediated activation of ERK (Figure 3A), JNK (Figure 3B), and p38 (Figure 3C). AP-1 composed of c-jun and c-fos acts as a nuclear transcription factor binds to MMPs promoter region and then induces MMPs gene transcription [4]. Treatment with GSE and GSC significantly inhibited phosphorylation of c-fos (Figure 4A) and c-jun (Figure 4B). Accordingly, GSE and GSC treatment inhibited UVB-mediated AP-1 overexpression.

### 3.5. Regulation of TGF-β and Smad Signaling

**Figure 5:**
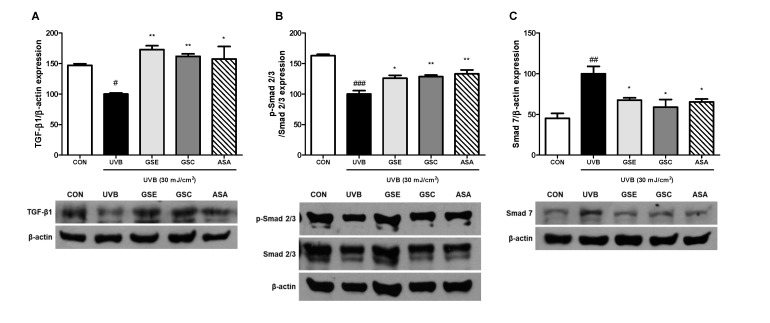
Effects of ginseng seed embryo (GSE) and coat (GSC) on transforming growth factor-β (TGF-β) level and phosphorylation of Smad. Control or UVB-irradiated cells were treated with/without GSE or GSC and subjected to western blotting for (**A**) TGF-β, (**B**) p-Smad 2/3, and (**C**) Smad 7 levels. The relative levels of the phosphorylated protein were normalized to those of β-actin or total Smad 2/3 proteins. Values are expressed as the mean ± standard error (*n* = 3). #, ##, and ### *p* < 0.05, 0.01, and 0.001, significant difference compared to control; * and ** *p* < 0.05 and 0.01, respectively, significant difference compared to UVB-irradiated group. ASA (ascorbic acid, 100 μM), positive control.

Previous studies have indicated that the TGF-β/Smad signaling pathway induces the expression of ECM proteins and inhibits their degradation by inhibiting MMPs [41,42]. To elucidate the mechanism of collagen synthesis and degradation, the effect of GSE and GSC treatment on the expression of TGF-β1, p-Smad 2/3, and Smad 7 in UVB-irradiated Hs68 cells were investigated. UVB irradiation significantly decreased the levels of TGF-β1 (Figure 5A) and p-Smad 2/3 (Figure 5B) by 46.99% and 62.93%, respectively, compared to the control cells. We further examined whether GSE and GSC treatment were involved in the regulation of Smad 7. UVB irradiation significantly increased Smad 7 expression by 54.69%, compared the control cells (Figure 5C). However, GSE and GSC treatment significantly downregulated the expression of Smad 7 proteins by 32.44% and 41.16% compared to the untreated with UVB-irradiated cells. These results indicate that ginseng seeds counteracted skin photoaging by reducing the protein levels of Smad 7 and improving Smad 2/3 and TGF-β expression.

## 4. Conclusions

Ginseng seeds contain high levels of functional compounds, including phytosterols, ginsenosides, vitamin E, and fatty acids that may contribute to their protective activity against photoaging in fibroblasts. Ginseng seeds mitigated ROS accumulation induced by UVB radiation. Stimulation of MMPs and downregulation of collagen by UVB was restored by GSE and GSC treatments, possibly through the modulation of collagen synthesis and degradation pathways. These results indicate that ginseng seeds protect human skin fibroblasts from UVB-induced photoaging and, therefore, has the potential for use in cosmeceutical preparations.

## Figures and Tables

**Table 1:** Antioxidant activities and phytochemical contents in ginseng seeds.

		GSE	GSC
*Antioxidant activities and compounds*			
DPPH radical scavenging (TE ^(1)^ mg/g residue)		6.96 ± 0.43	16.02 ± 2.81
ABTS radical scavenging (TE mg/g residue)		17.35 ± 0.33	49.34 ± 0.89
Total polyphenolic contents (GAE ^(2)^ mg/g residue)		4.37 ± 0.04	5.87 ± 0.07
Total flavonoid contents (CE ^(3)^ mg/g residue)		1.62 ± 0.05	2.70 ± 0.04
*Phytosterols (mg/g residue)*			
Campesterol		6.35 ± 0.10	6.11 ± 0.41
Stigmasterol		15.62 ± 0.60	2.96 ± 0.55
β-sitosterol		20.51 ± 0.57	4.01 ± 0.35
*Ginsenosides (mg/g residue)*			
Rg_1_		0.04 ± 0.01	0.69 ± 0.00
Re		0.81 ± 0.10	1.22 ± 0.09
Rk_3_		N.D. ^(4)^	0.45 ± 0.12
Rh_2_		0.26 ± 0.05	N.D.
Rh_4_		0.80 ± 0.04	10.45 ± 0.16
*Tocopherols and tocotrienol (μg/g residue)*			
α-Tocopherol		30.19 ± 0.22	N.D.
α-Tocotrienol		202.97 ± 3.66	12.14 ± 0.56
γ-Tocopherol		50.74 ± 0.05	15.88 ± 1.08
*Fatty acids (mg/g residue)*			
Lauric acid	12:0	N.D.	0.14 ± 0.00
Myristic acid	14:0	N.D.	0.19 ± 0.00
Palmitic acid	16:0	5.28 ± 0.24	3.15 ± 0.04
Hexadecenoic acid	16:1	0.14 ± 0.00	0.66 ± 0.03
Stearic acid	18:0	0.35 ± 0.01	0.64 ± 0.01
Oleic acid	18:1(n-9)	6.74 ± 0.36	9.88 ± 0.48
Vaccenic acid	18:1(n-7)	0.98 ± 0.06	1.64 ± 0.05
Linolelaidic acid	18:2t	0.25 ± 0.03	0.41 ± 0.02
Linoleic acid	18:2(n-6)	19.33 ± 0.96	11.50 ± 0.55
Linolenic acid	18:3t	N.D.	0.42 ± 0.02
Linolenic acid	18:3(n-3)	0.33 ± 0.01	0.15 ± 0.01
Eicosanoic acid	20:1	0.11 ± 0.01	N.D.
	Total	33.50 ± 1.62	28.88 ± 1.19

Values are the mean ± standard error of at least two experiments. The yield of Ginseng seed embryo (GSE) and ginseng seed coat (GSC) was 11.38% and 2.77%, respectively. ^(1)^ Trolox equivalents ^(2)^ Gallic acid equivalents ^(3)^ Catechin equivalents ^(4)^ Not detected (N.D.).

## Data Availability

Not applicable.

## Supplementary Materials

The following are available online at https://www.mdpi.com/2076-3921/10/3/403/s1, Figure S1: phytosterols chromatogram in standards (A), ginseng seed embryo (B), and ginseng seed coat (C) by GC analysis. Figure S2: tocopherols and tocotrienols chromatogram in standards (A), ginseng seed embryo (B), and ginseng seed coat (C) by HPLC analysis. T, tocopherol; T3, tocotrienol. Figure S3: ginsenosides chromatogram in standards (A), ginseng seed embryo (B), and ginseng seed coat (C) by HPLC analysis. Figure S4: fatty acids chromatogram in standards (A), ginseng seed embryo (B), and ginseng seed coat (C) by GC analysis.
