# Supplementary material for: Protective Activity and Underlying Mechanism of Ginseng Seeds against UVB-Induced Damage in Human Fibroblasts
Source: Antioxidants (Basel). 2021 Mar 8;10(3):403. doi: 10.3390/antiox10030403 (PMC8001990; doi:10.3390/antiox10030403)
Supplement: Supplementary file 1 [file antioxidants-10-00403-s001.pdf]

Supplementary Materials

Protective Activity and Underlying Mechanism of Ginseng Seeds against UVB-induced Damage in Human Fibroblasts

Huijin Heo[1], Hana Lee[1], Jinwoo Yang[2], Jeehye Sung[3], Younghwa Kim[4], Heon Sang Jeong[1], Junsoo Lee[1,*]

[1] Department of Food Science and Biotechnology, Chungbuk National University, Cheongju, Chungbuk 28644, Republic of Korea; pltreasure11@gmail.com (H.H.); dlgksk0514@naver.com (H.L.); hsjeong@chungbuk.ac.kr (H.J.); junsoo@chungbuk.ac.kr (J.L.)

[2] Wheat Research Team, National Institute of Crop Science, Rural Development Adminstration, Wanju, Jeonbuk 55365, Republic of Korea; jinwoo1127@korea.kr

[3] Department of Food Science and Biotechnology, Andong National University, Andong, Gyeongbuk 36729, Republic of Korea; jeehye@anu.ac.kr

[4] School of Food Biotechnology and Nutrition, Kyungsung University, Busan, 48434, Republic of Korea; younghwakim@ks.ac.kr

(A) Phytosterol standards

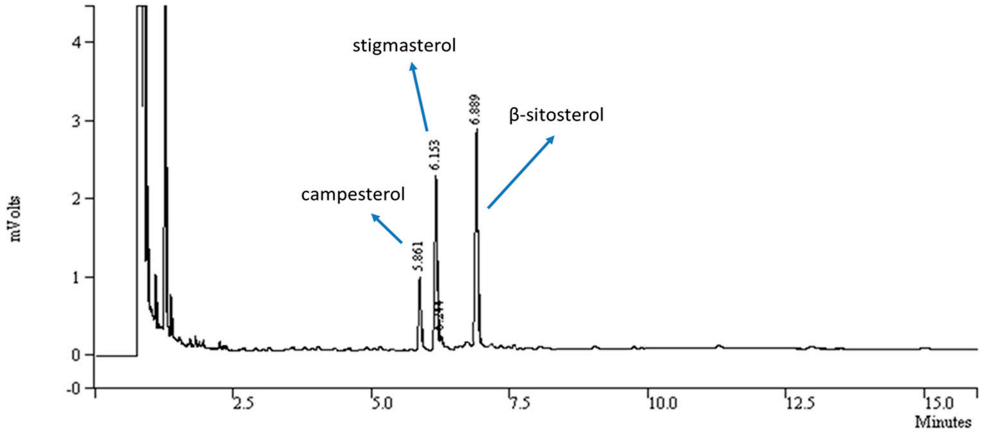

(B) Ginseng seed embryo (GSE)

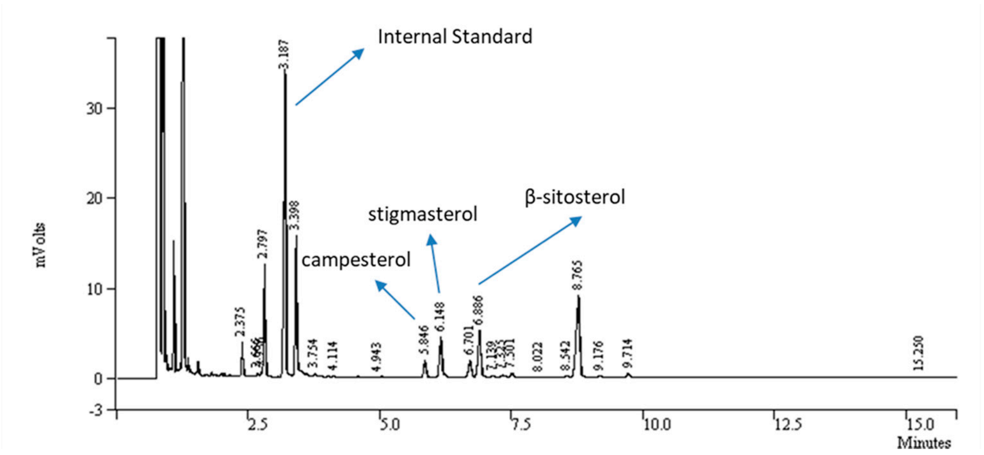

(C) Ginseng seed coat (GSC)

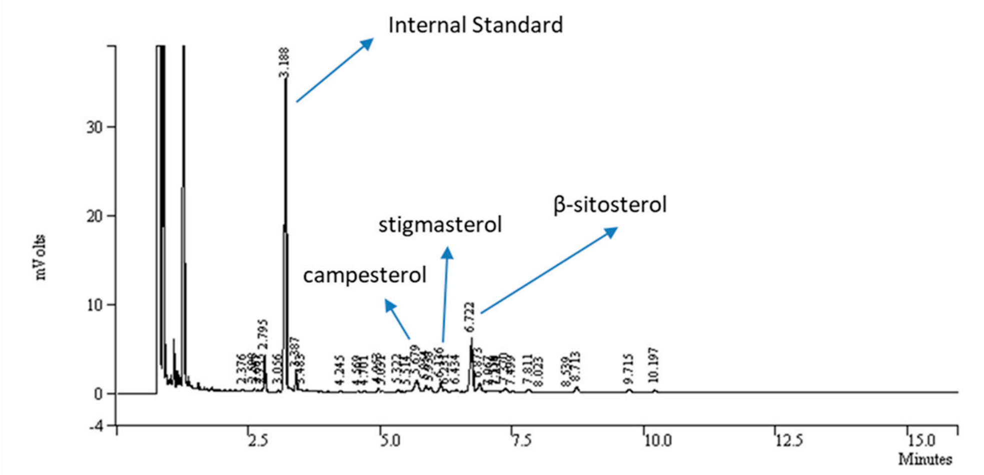

Figures S1. Chromatogram of phytosterols in standards (A), ginseng seed embryo (B), and ginseng seed coat (C) by GC analysis.

(A) Tocopherol and tocotrienol standards

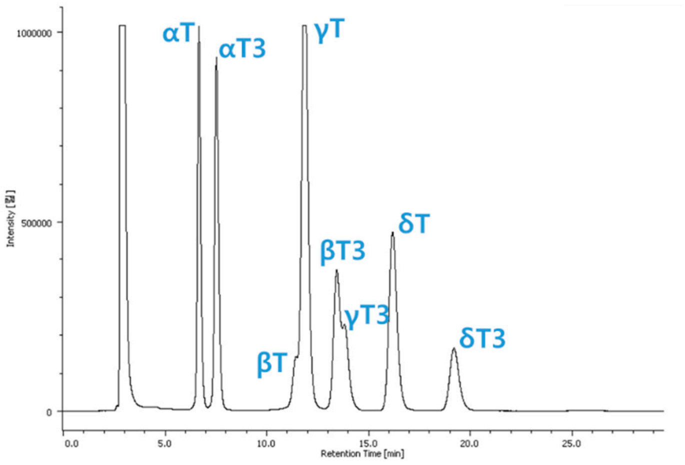

(B) Ginseng seed embryo (GSE)

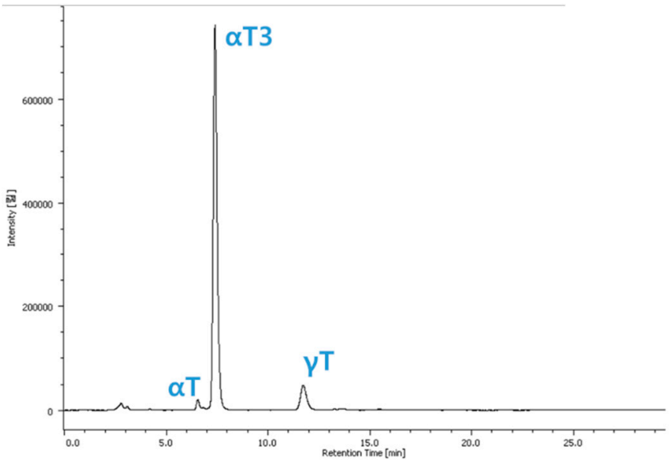

(C) Ginseng seed coat (GSC)

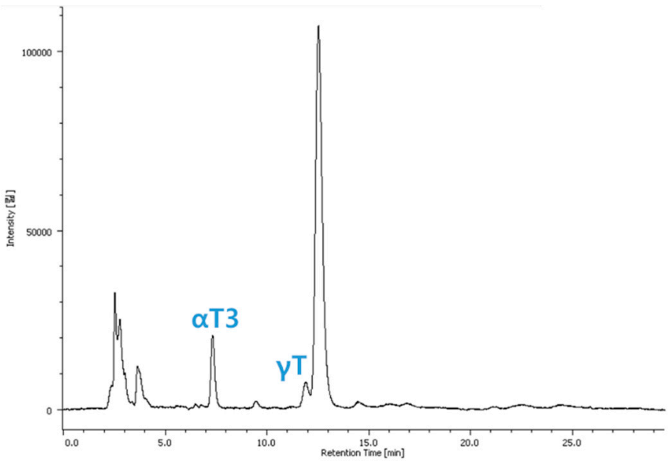

Figures S2. Chromatogram of tocopherols and tocotrienols in standards (A), ginseng seed embryo (B), and ginseng seed coat (C) by HPLC analysis. T, tocopherol; T3, tocotrienol.

(A) Ginsenoside standards

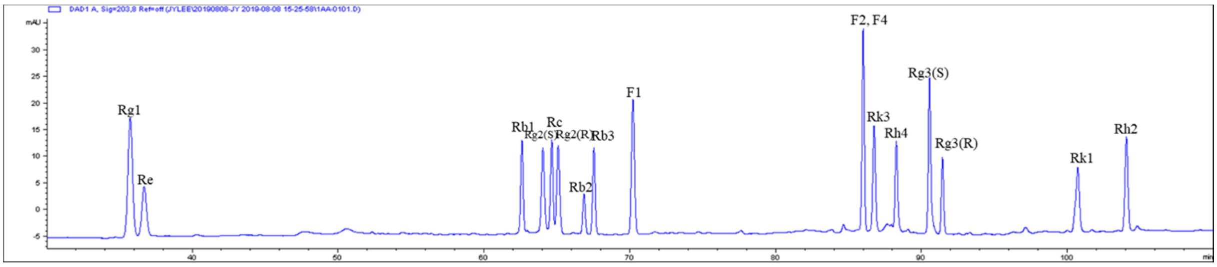

(B) Ginseng seed embryo (GSE)

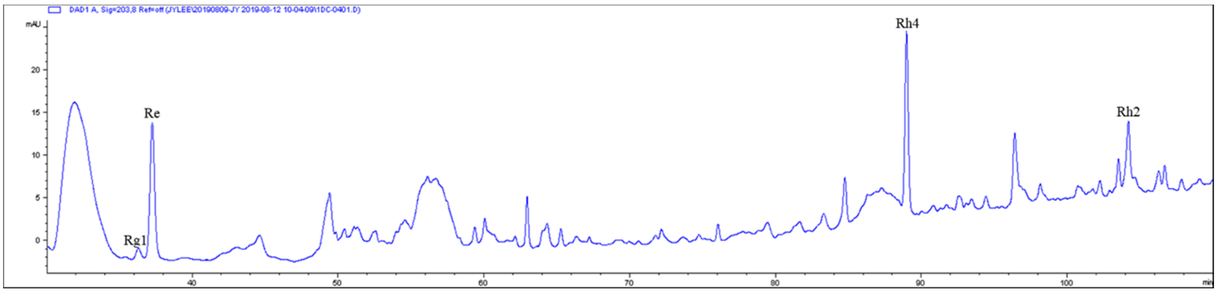

(C) Ginseng seed coat (GSC)

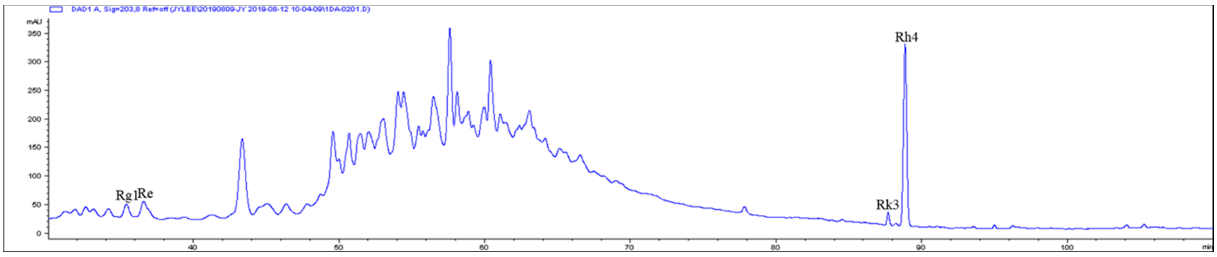

Figure S3. Chromatogram of ginsenosides in standards (A), ginseng seed embryo (B), and ginseng seed coat (C) by HPLC analysis.

(A) Fatty acid standards

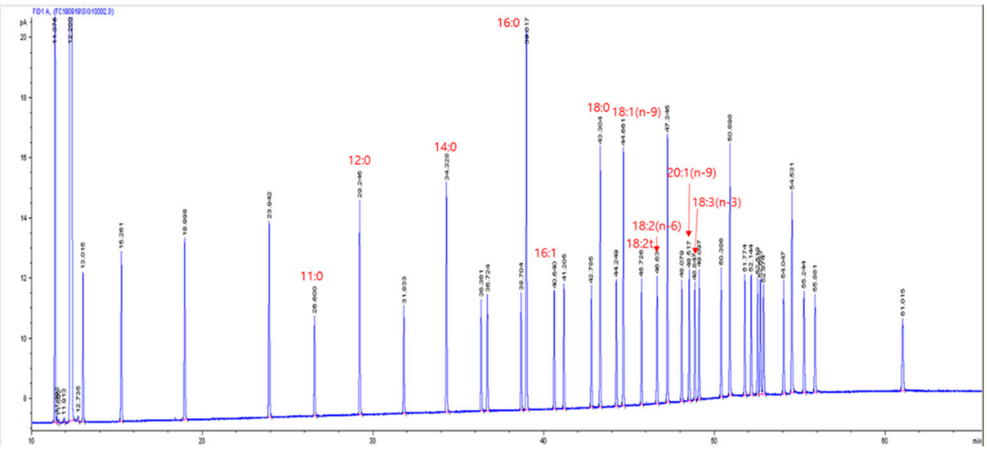

(B) Ginseng seed embryo (GSE)

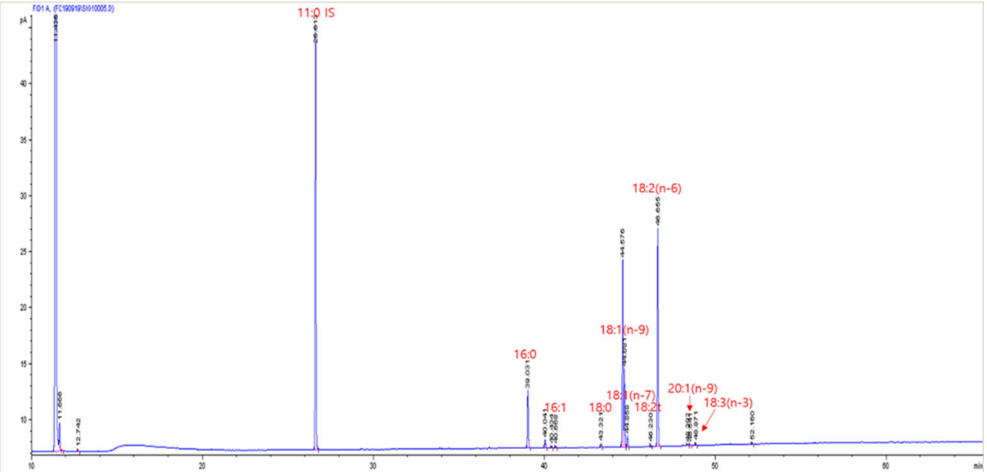

(C) Ginseng seed coat (GSC)

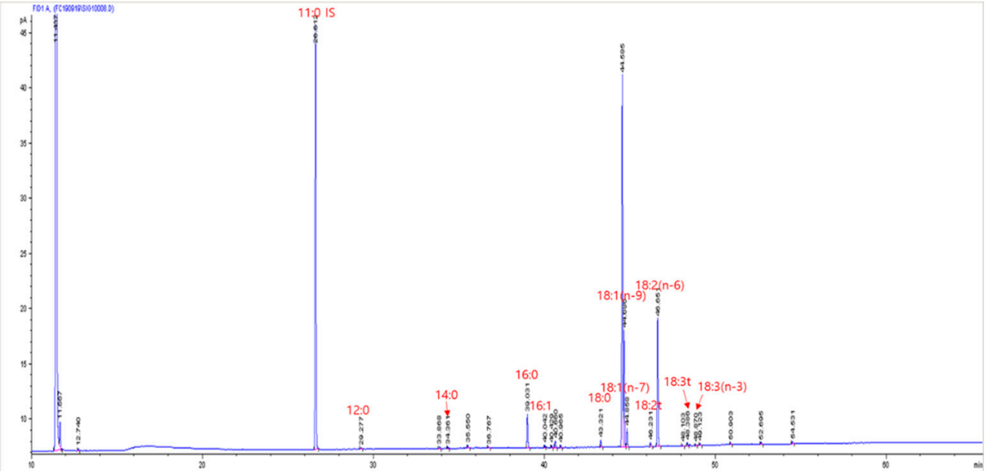

Figure S4. Chromatogram of fatty acids in standards (A), ginseng seed embryo (B), and ginseng seed coat (C) by GC analysis.
